# Molecular autopsy by trio exome sequencing (ES) and postmortem examination in fetuses and neonates with prenatally identified structural anomalies

**DOI:** 10.1038/s41436-018-0298-8

**Published:** 2018-10-08

**Authors:** Elizabeth Quinlan-Jones, Jenny Lord, Denise Williams, Sue Hamilton, Tamas Marton, Ruth Y. Eberhardt, Gabriele Rinck, Elena Prigmore, Rebecca Keelagher, Dominic J. McMullan, Eamonn R. Maher, Matthew E. Hurles, Mark D. Kilby

**Affiliations:** 1 Department of Clinical Genetics, Birmingham Women’s & Children’s NHS Foundation Trust, Birmingham, UK; 2 West Midlands Fetal Medicine Centre, Birmingham Women’s & Children’s NHS Foundation Trust, Birmingham, UK; 3 Wellcome Sanger Institute, Hinxton Cambridge, UK; 4 West Midlands Regional Genetics Service, Birmingham Women’s and Children’s Hospital NHS Foundation Trust, Birmingham, UK; 5 West Midlands Regional Perinatal Pathology Service, Birmingham Women’s and Children’s NHS Foundation Trust, Birmingham, UK; 6 Department of Medical Genetics, University of Cambridge and NIHR Cambridge Biomedical Research Centre, Cambridge, UK; 7 Institute of Metabolism and Systems Research, College of Medical and Dental Sciences, University of Birmingham, Birmingham, UK

**Keywords:** exome sequencing, fetuses, neonates, autopsy, genetic diagnosis

## Abstract

**Purpose:**

To determine the diagnostic yield of combined exome sequencing (ES) and autopsy in fetuses/neonates with prenatally identified structural anomalies resulting in termination of pregnancy, intrauterine, neonatal, or early infant death.

**Methods:**

ES was undertaken in 27 proband/parent trios following full autopsy. Candidate pathogenic variants were classified by a multidisciplinary clinical review panel using American College of Medical Genetics and Genomics (ACMG) guidelines.

**Results:**

A genetic diagnosis was established in ten cases (37%). Pathogenic/likely pathogenic variants were identified in nine different genes including four de novo autosomal dominant, three homozygous autosomal recessive, two compound heterozygous autosomal recessive, and one X-linked. *KMT2D* variants (associated with Kabuki syndrome postnatally) occurred in two cases. Pathogenic variants were identified in 5/13 (38%) cases with multisystem anomalies, in 2/4 (50%) cases with fetal akinesia deformation sequence, and in 1/4 (25%) cases each with cardiac and brain anomalies and hydrops fetalis. No pathogenic variants were detected in fetuses with genitourinary (1), skeletal (1), or abdominal (1) abnormalities.

**Conclusion:**

This cohort demonstrates the clinical utility of molecular autopsy with ES to identify an underlying genetic cause in structurally abnormal fetuses/neonates. These molecular findings provided parents with an explanation of the developmental abnormality, delineated the recurrence risks, and assisted the management of subsequent pregnancies.

## Introduction

Fetal structural anomalies (FSAs) complicate 3% of pregnancies and range in phenotype from isolated minor anomalies to severe multisystem abnormalities, many of which are associated with high perinatal mortality rates, or contribute to long-term morbidity.^1^ Currently, such pregnancies may be investigated prenatally by chorionic villus sampling (CVS) or amniocentesis, to obtain fetal DNA for quantitative fluorescent–polymerase chain reaction (QF-PCR) to exclude common autosomal and sex chromosome aneuploidies, and chromosomal microarray (CMA) to detect submicroscopic copy-number variations (CNVs).^2,3^ Adding CMA analysis to G-banded karyotyping increases the detection rate in this group of fetuses by up to 5% (ref. ^4^). However, no identifiable genetic cause is detected in over 60% of cases.^5^ If the pregnancy is terminated or fetal demise occurs, autopsy is important because despite generally good correlation between prenatal ultrasound and autopsy findings, the latter can identify additional subtle malformations and may elucidate further information allowing an etiologic diagnosis necessary for accurate genetic counseling.^6,7^

Exome sequencing (ES) is an established diagnostic tool in delineating the genetic etiology of congenital abnormalities and neurodevelopmental disorders^8,9^ in adults and children. The use of ES for the analysis of prenatally obtained fetal DNA has been reported in small cohort series and a recent review has indicated that this technology reveals pathogenic findings in a range of between 6.2% and 80% of cases.^10^ Our group has reported prospective data on 610 prenatal cases with a spectrum of fetal anomalies detected by ultrasound scan (USS) and demonstrated a diagnostic yield of 8.5% (95% confidence interval [CI]: 6.4–11.0%); a further 3.9% had a variant of uncertain significance (VUS) with potential clinical value.^11^

One of the limitations in the use of prenatal ES is a lack of accurate fetal phenotyping using prenatal ultrasonography alone.^10^ In the cases of FSA ending in fetal demise, postnatal dysmorphological examination and autopsy in addition to prenatal ultrasound might be predicted to improve the interpretation of genetic findings and increase diagnostic yield. We have evaluated this in a cohort of fetuses/neonates (*n* = 27). We correlated the results of ES from proband–parent trios with prenatal USS and autopsy findings to determine the clinical utility of molecular autopsy. Our study supports the use of trio ES and autopsy to identify the underlying etiology of structural anomalies, expand knowledge of the genetic basis of fetal development, and enable improved descriptions of phenotypic variation of known genetic disorders.^12^

## Materials and methods

### Recruitment of cases

Parents of fetuses/neonates (*n* = 27) with a significant structural anomaly resulting in termination of pregnancy (TOP), intrauterine fetal demise (IUFD), or neonatal/infant death (NND/ID) were identified by clinical geneticists, perinatal pathologists, and fetal medicine specialists at Birmingham Women’s and Children’s NHS Foundation Trust (BWCNFT) and recruited to this study between May 2015 and December 2017. Prospectively obtained fetal DNA at autopsy was stored at West Midlands Regional Genetics Laboratory (WMRGL) and, with written consent, trio (proband/biparental) ES was performed at the Wellcome Sanger Institute (WSI) in Cambridge, United Kingdom. All cases had previously undergone standard testing for aneuploidy (QF-PCR) and whole-genome copy-number analysis by CMA with no pathogenic abnormalities found. Parents were prospectively informed that only results relevant to the USS-detected FSA would be reported back (i.e., not secondary findings). Ethics approval to undertake the research was granted by National Research Ethics Service (NRES) Committee West Midlands–South Birmingham (ref: 13/WM/1219). NHS Trust approval was provided by BWCNFT–Research and Development Department.

### Exome sequencing, variant calling, and annotation

In 26 cases DNA was extracted directly from fetal tissue obtained at autopsy, and in one case fetal DNA was extracted from cultured amniocytes obtained prenatally. The autopsy tissue used to extract DNA varied from case to case and included skin, lung, liver, and muscle. Parental DNA was obtained from blood or saliva samples to form proband/parent trios. Trio DNA samples were batched at WMRGL and transferred to the WSI (Cambridge, UK) for ES through the PAGE Study^13^ sequencing pipeline. For full information on sequencing and analysis see Supplementary Methods. Briefly, 125 ng genomic DNA was fragmented (~150 bp) and library preparation was conducted following Illumina’s standard methodologies. Indexed libraries were pooled and exome capture undertaken with the Agilent SureSelect XT Human All Exon V5 Plus with custom ELID#0337431 (Agilent Technologies, Santa Clara, CA, USA). Seventy-five base paired-end sequencing (with six samples per lane on Illumina HiSeq 2500) was conducted following the manufacturer’s instructions, with over 97% of exonic positions having depth of coverage >13×. Mapping was conducted with the Burrows–Wheeler Aligner (BWA, version 0.59). Single-nucleotide variants (SNVs) and indels were identified using GATK HaplotypeCaller version 3.6 (ref. ^14^), while DeNovoGear^15^ was used to identify de novo variants, and CoNVex (http://www.uk10k.org/assets/ashg_vijayarangakannan_etal_2012.pdf) and CIFER (https://github.com/jeremymcrae/cifer) were used to identify and assess the inheritance of CNVs. Variant call format (VCF) files were annotated using Ensembl’s Variant Effect Predictor (VEP).^16^ Variants were filtered to identify those of potential clinical significance based on functional consequence (i.e., protein altering variants) and minor allele frequency (MAF) across a number of resources (see Supplementary Methods) (MAF > 0.005 excluded, with more stringent filters applied for variants in monoallelic genes [MAF >0.0005 excluded if both parents' data present]). The analysis pipeline produced a selection of candidate pathogenic variants with an inheritance pattern consistent with diseases associated with the gene in question, and were filtered using a modified developmental disorder–associated gene panel. A virtual gene panel was used to maximize diagnostic yield and minimize the potential for secondary findings, and high numbers of variants of uncertain significance. This modified gene panel included the full DDG2P gene list^17^ (https://www.ebi.ac.uk/gene2phenotype) with several genes removed due to the absence of a prenatal phenotype, as well as some additional genes known to be associated with fetal development sourced from the literature (see Supplementary Table 1). Sequencing data is available from the European Genome-phenome Archive (https://www.ebi.ac.uk/ega/).

### Variant interpretation and classification

A multidisciplinary clinical review panel (CRP) comprising clinical geneticists, fetal medicine specialists, perinatal pathologists, clinical scientists, midwives, and bioinformaticians reviewed and classified candidate pathogenic variants.^11^ Through consensus agreement variants were classified using the American College of Medical Genetics and Genomics (ACMG) guidelines.^18^ Pathogenic and likely pathogenic variants were further classified as to whether they explained the observed phenotype fully, partially, or did not explain the phenotype.^18^ The autopsies were carried out at West Midlands Regional Perinatal Pathology Service (located within a tertiary, teaching hospital setting) by designated subspecialty perinatal pathologists after informed consent was sought from parents. In all cases a full autopsy was performed according to Royal College of Pathologists guidelines,^19,20^ which included external examination, X-ray, comprehensive internal examination, and histology of relevant internal organs. Abnormal findings were photographically documented and discussed with senior clinical geneticists at a regular dysmorphology meeting. Pathogenic/likely pathogenic variants assessed to be causative for the observed fetal phenotype were confirmed by Sanger sequencing. Reports were issued to the clinical geneticist/fetal medicine specialist involved in the care of the family to return the findings.

### Statistical analysis

Fisher’s exact test was used to compare the diagnostic rates and the number of homozygous autosomal recessive diagnoses between cases with and without self-reported consanguinity using R (version 3.1.3).

## Results

### Demographic characteristics

The median age of women recruited was 30 years (95% CI 27.4–31.4). Nine (33%) women were nulliparous and 18 (67%) were multiparous. Gestational age (GA) at delivery ranged from 15 to 35 weeks (median GA 23 weeks) (95% CI 21.4–28.8). In five pregnancies (19%) the women had a previous history of a baby with a congenital malformation and in six pregnancies (22%) there was parental consanguinity. There were 15 (55%) TOP, 8 (30%) IUFD, and 4 (15%) NND/ID. Gestational age ranged from 15 to 35 weeks (TOP), 16 to 34 weeks (IUFD), and 0 to 58 days postnatal (NND/ID). There was no correlation between the presence of a diagnostic ES finding and maternal age, ethnicity, parity, or GA. Pregnancy outcome per proband according to phenotype class (determined at postmortem [PM] examination) is presented in Supplementary Figure 1.

### Phenotype classification

Phenotypic information was obtained from the prenatal imaging (USS and fetal magnetic resonance image [MRI]) reports, and from the final autopsy report. Initially, the 27 probands (10 female, 17 male) were categorized by phenotype into 12 classes according to the anatomical system affected, jointly by the fetal medicine specialist and clinical geneticist at the study site. Following this, the perinatal pathologist also independently categorized the cases according to the same criteria (Tables 1 and 2). This resulted in the reclassification of 4 fetal phenotypes (15%), increasing the number of brain (1 to 3) and abdominal (0 to 1) phenotypes, and reducing the number of fetal akinesia deformation sequence (FADS) (4 to 3), genitourinary (2 to 1), and multisystem (14 to 13) phenotypes; the cases of cardiac (4), skeletal (1), and hydrops fetalis (1) remained unchanged. Reclassification of four cases by the perinatal pathologist according to the autopsy findings occurred for the following indications. Case 12 (see Table 2) was an isolated omphalocele with no associated ventriculomegaly (VM) noted at autopsy and was therefore reclassified as an isolated abdominal malformation. Prenatally it was reported that the lateral ventricles measured just over 10 mm bilaterally inferring “borderline” VM as an ultrasound finding. It is possible that the VM was present and subsequently resolved with advancing gestation. However, it has been described that autopsy may fail to confirm VM identified prenatally in approximately 50% of cases due to resolution rather than autopsy artifact.^21^ Case 15 had a brain abnormality identified prenatally (see Table 2), and subtle skull, facial, and cardiac abnormalities confirmed at autopsy. It was concluded that the additional anomalies observed postnatally were subsequent to the primary brain anomaly and thus the phenotype was reclassified from multisystem to brain. The autopsy examination for case 19 (see Table 2) identified internal genital and vertebral anomalies in addition to the renal malformations diagnosed on prenatal ultrasound, and hence it was reclassified from renal to multisystem. Finally in case 23 (see Table 2) the observed FADS was determined as having been caused by an underlying central nervous system abnormality and was therefore reclassified to the brain abnormality group. Almost half (13 cases) were classified as having structural anomalies affecting multiple organ systems. The remaining were classified as cardiac (4), FADS (3), brain (3), genitourinary (1), skeletal (1), abdominal (1), and hydrops fetalis (1) with no other structural abnormality. There were no cases with isolated facial, chest, or spinal anomalies, or raised nuchal translucency (NT) >4 mm (i.e., the minimum measurement required a priori to be eligible for enrollment in the study).

**Table 1:** Exome sequencing (ES) diagnostic cases

Patient number	Prenatal USS findings	PM findings	Phenotypic classification based on USS and PM	Gene	Alteration	Inheritance	Zygosity	Associated clinical condition	ACMG classification (Richards, 2015)
3	Univentricular heart, TGA, HLHS	Mitral atresia, DORV, univentricular heart, cleft palate, horse kidney, hypoplastic gall bladder, pulmonary hypoplasia, facial dysmorphia	Multisystem	***KMT2D***	c.3249C>A p.(Cys1083*)	De novo	Heterozygous	**Kabuki syndrome**	**Pathogenic** (PM2, PSV1, PS2)
8	FADS	Arthrogryposis multiplex, clenched hands with overlapping fingers, skin edema, high arched palate	FADS	***RAPSN***	c.485A>G p.(Glu162Gly)	Inherited	Homozygous	**Congenital myasthenic syndrome/multiple pterygium syndrome**	**Likely pathogenic** (PM2, PP3, PM5, PP4**)**
9	FADS	Arthrogryposis multiplex, overlapping fingers with abducted thumbs, high arched palate, pulmonary hypoplasia, cerebellar hypoplasia, hydrops fetalis	FADS	***ERCC5***	c.2766dupA p.(leu923Thrft*7)	Inherited	Homozygous	**Cerebro-oculofacioskeletal syndrome (COFS)**	**Pathogenic** (PM2, PS4_moderate, PSV1, PP1)
10	Cystic hygroma (>4.0 mm), hydrops fetalis	Large cystic hygroma, hydrops fetalis, persistent left SVC, ASD, bilateral talipes	Multisystem	***RIT1***	c.268A>G p.(Met90Val)	De novo	Heterozygous	**RIT1-associated Noonan syndrome**	**Pathogenic** (PM2, PS4_moderate, PP3, PM5, PS2)
11	Nuchal translucency measurement 8.4 mm	Atrial ventricular channel, DORV, right-sided aortic arch, aortic valve atresia, pulmonary hypoplasia, nuchal edema	Cardiac	***DNAH5***	c.6763C>T p.(Arg2255*) c.13458dup p.(Asn4487*)	Inherited (maternally and paternally)	Compound heterozygous	**Primary ciliary dyskinesia (PCD)**	**Pathogenic (**c.6763C>T) (PM2, PVS1, PS4_moderate) **Pathogenic** (c.13458dup) (PM2, PVS1, PM3)
16	Hydrops fetalis	Hydrops fetalis, flexed upper limbs, talipes, interrupted aortic arch, unilateral renal agenesis with contralateral renal dysplasia	Multisystem	***PIGN***	c.548_549+delAGGTTTGT p.?AR c.654T>G p.(His218Gln)	Inherited (maternally and paternally)	Compound heterozygous	**PIGN-associated epilepsy/multiple congenital anomalies–hypotonia–seizures syndrome**	**Pathogenic** (c.548_549+delAGGTTTGT) PM2,PVS1,PM3 **Variant of uncertain significance (VUS)** (c.654T>G) (PM2, PP3, PM3)
21	Hydrops fetalis	Hydrops fetalis, bilateral talipes, pulmonary hypoplasia	Hydrops fetalis	***FOXP3***	c.1189C>T p.(Arg397Trp)	Inherited (maternally)	Hemizygous	**IPEX syndrome in males**	**Likely pathogenic** (PM2, PS4_moderate, PP3, PS3)
22	Hydrops fetalis	Hydrops fetalis, cystic hygroma, micrognathia, cleft palate, TGA, persistent SVC, VSD, bilateral dysplastic kidneys	Multisystem	***KMT2D***	c.1434G.T p.(Glu4781*)	De novo	Heterozygous	**Kabuki syndrome**	**Pathogenic** (PM2, PVS1, PS2_supporting)
24	Bilateral ventriculomegaly and lissencephaly	Facial dysmorphia, polymicrogyria of CNS and adrenal cytomegaly	Brain	***PIK2R2***	c.1117G>A p.(Gly373Arg)	De novo	Heterozygous	**Megalencephaly–polymicrogyria–polydactyly–hydrocephalus syndrome (MPPH)**	**Pathogenic** (PM2, PS2, PS3_mod, PS4, PP3)
25	Renal cystic dysplasia	Facial dysmorphia, polymicrogyria of CNS with calcification, bilateral renal cystic dysplasia	Multisystem	***CPT2***	c.28_29insAGCAAG p.(Try10*)	Inherited	Homozygous	**Carnitine palmitoyltransferase II deficiency**	**Likely pathogenic** (PVS1, PM2)

*ACMG* American College of Medical Genetics and Genomics, *ASD* atrial septal defect, *CNS* central nervous system, *DORV* double outlet right ventricle, *FADS* fetal akinesia deformation sequence, *HLHS* hypoplastic left heart syndrome, *PM* postmortem, *SVC* superior vena cava, *TGA* transposition of the great arteries, *USS* ultrasound scan, *VSD* ventricular septal defect, Patient number = chronological patient number, Prenatal USS finding = Prenatal ultrasound findings, PM findings = Post-mortem findings, Gene. The gene is noted in italics.

**Table 2:** Exome sequencing (ES) nondiagnostic cases

Proband ID	Prenatal USS findings	PM findings	Phenotype classification based upon USS and PM
1	NT measurement 4.0 mm, arthrogryposis	Skin webs, flexed contractures, severe bilateral talipes, webbed neck, micrognathia, reduced muscle bulk, subcutaneous edema	FADS
2	Asymmetrical ventriculomegaly (displacement of the midline), irregularity of the ventricular lining (possibly neuronal heterotopias), possibly absent corpus callosum, abnormal posterior fossa, single outlet to the heart, VSD	Large cranial vault, TOF, persistent left SVC, right-sided aortic arch, absent right umbilical artery, abnormally shaped thymus, severe dysplasia of the cerebellum with obliteration of the 4th ventricle, malformed brainstem and midbrain, atresia of aqueduct of Sylvius and 4th ventricle, enlarged right cerebral hemisphere, probable arachnoid cyst between hemispheres, abnormal deep white matter bundles in cerebral hemispheres	Multisystem
4	Short long bones and talipes	Small size, facial dysmorphia, prominent occiput, bilateral talipes, rhizomelic shortening of the limbs, wide-set nipples, osteopenic bones, horizontal ribs	Skeletal
5	Hydrops fetalis	Facial dysmorphia, nuchal edema, dilated left atrium and left ventricle, cardiomegaly, valvular aortic atresia	Cardiac
6	Small left ventricle, bilateral superior vena cavas, levocardia with significant ventricular imbalance, small slit-like left ventricle with some mitral inflow, perimembranous VSD, normal large vessels, HLHS Ebstein-type abnormality, DORV, abnormal left kidney	Overlapping fingers, bilateral talipes, dysmorphic ears, skin webbed neck, cardiomegaly, pulmonary stenosis with dysplastic valves, VSD, right ventricular pouch, persistent left SVC, small testis/thymus, ectopic left kidney	Multisystem
7	Polyhydramnios, micrognathia, right-side mild dilatation of the renal pelvis	Facial dysmorphia, hypoplastic lungs, CPAM-0, small gall bladder, small right kidney with dilated renal pelvis, simple renal cysts bilaterally, gracile bones (especially the clavicles, fracture of the right clavicle), excess of extramedullary hematopoiesis in the liver	Multisystem
12	Exomphalos, borderline ventriculomegaly	Omphalocele, bilateral talipes	Abdominal
13	Bilateral multicystic, dysplastic kidneys	Facial dysmorphia, pterygium of the neck, contractures of the upper limbs, bilateral talipes, transverse palmar creases, bilateral cystic–dysplastic kidneys, lung hypoplasia	Genitourinary
14	Growth restriction <10th centile	Retrognathia, ASD (secundum type), small thymus, lung petechiae, distended bladder with retained urine (no evidence of obstruction)	Cardiac
15	Unilateral ventriculomegaly	Macrocephaly, partly compressed skull, overlapping cranial bones, loose scalp, anteverted nares, small heart	Brain
17	Normal	Micrognathia, omphalocele, bilateral talipes, bilaterally three lobed lungs, irregularly shaped left kidney, cervical ribs	Multisystem
18	Long narrow chest, heart in unusual orientation, single multicystic dysplastic kidney, unilateral talipes, umbilical vein varix, minimal liquor	Facial dysmorphia, left-side talipes, caudal orientation of the heart, aortic valve dysplasia, rectal atresia, multicystic/aplastic renal dysplasia, lumbar and sacral vertebrae defects	Multisystem
19	Oligohydramnios, unilateral talipes,?absent bladder and kidneys, IUGR	Potter syndrome, bilateral talipes, malpositioned anus, transverse palmar creases (right hand), epicanthus, renal agenesis, unicornuate uterus, lung hypoplasia, irregularities of the sacrum vertebrae	Multisystem
20	Normal	Unilateral talipes, complete AV channel, DORV, pulmonary stenosis, symmetrical liver, absent gall bladder, right type isomerism of the lungs, auricles, absent spleen, right-sided stomach	Multisystem
23	Polyhydramnios	Abnormal posture of limbs (flexed legs in the hip and pointy feet), ulnar deviation of the hands and flexed thumbs, thin neck and diaphragm, lung hypoplasia, thin ribs, Arnold–Chiari type II (no cerebellar hypoplasia or spina bifida)	Brain
26	Congenital bladder neck obstruction	Subcutaneous edema, scoliosis, talipes and skeletal abnormalities, distended abdominal wall, anal atresia, abnormal genitalia, bladder neck obstruction with cystic bladder, rectal–vesical fistula, small lungs, small kidneys and adrenals, cystic–dysplastic change in kidneys associated to LUTO	Multisystem
27	HLHS	HLHS, 11 pairs of ribs, low-set eyes	Cardiac

*ASD* atrial septal defect, *AV* atrioventricular, *CPAM* congenital pulmonary airway malformation, *DORV* double outlet right ventricle, *FADS* fetal akinesia deformation sequence, *HLHS* hypoplastic left heart syndrome, *IUGR* intrauterine growth restriction, *LUTO* lower urinary tract obstruction, *NT* nuchal translucency, *PM* postmortem, *SVC* superior vena cava, *TOF* tetralogy of Fallot, *USS* ultrasound scan, *VSD* ventricular septal defect.

### Variant assessment

**Fig. 1:**
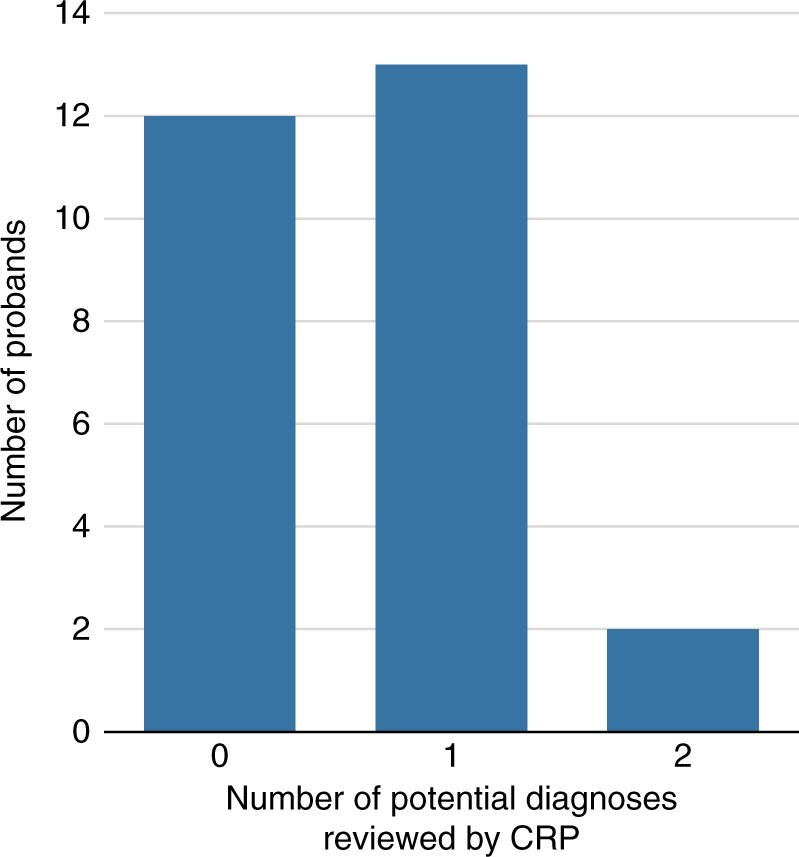
**Number of potential clinical diagnoses per proband reviewed by the clinical review panel (CRP).**

After bioinformatic filtering, 22 rare variants relating to 17 potential diagnoses in 15 probands were reviewed by the CRP. In the remaining 12 individuals no candidate pathogenic variants were identified. The mean number of variants identified per case was 1.47 (range 1–3) for the 15 cases with variants to review, and 0.815 across the entire cohort of 27 (Fig. 1). Variants were assessed in 16 different genes known to be associated with various developmental disorders. Of the 15 cases, 13 had variants to review in a single gene (heterozygous [6], compound heterozygous [4], or homozygous [3]) and 2 fetuses harbored homozygous or compound heterozygous variants in two different genes.

### Pathogenic variants

**Fig. 2:**
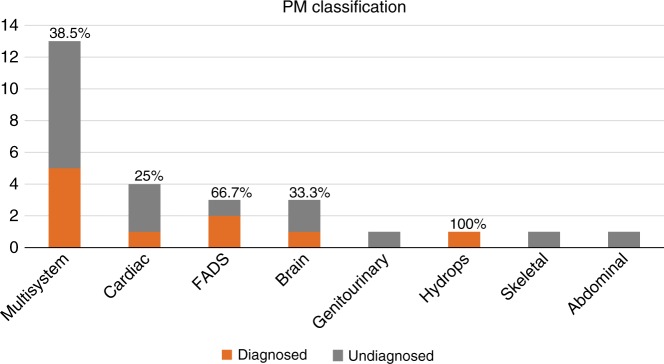
**Diagnostic (pathogenic/likely pathogenic) variant rate according to phenotype classification determined at postmortem examination.** *FADS* fetal akinesia deformation sequence, *PM* postmortem.

Of the 15 fetuses reviewed by the CRP, 10 were found to be harboring pathogenic/likely pathogenic variants determined to have fully/partially contributed to the phenotype observed on prenatal USS and autopsy, giving a diagnostic rate of 37% (Table 1). Four of the ten pathogenic variants had arisen de novo (two cases with protein truncating variants in *KMT2D*, and one case each with missense variants in *PIK3R2* and *RIT1*). The remaining pathogenic variants were inherited, including a maternally inherited *FOXP3* missense variant in a male fetus, and three cases with recessively inherited homozygous variants (including a missense variant in *RAPSN*, and protein truncating variants in *ERCC5* and *CPT2)*. The final two cases had biparentally inherited compound heterozygous variants (a paternally inherited missense variant and a maternally inherited protein truncating variant in *PIGN*, and two different biparentally inherited protein truncating variants in *DNAH5*). The diagnostic yield across different phenotypic classes is presented in Fig. 2 (based on PM classification). *KMT2D* gene alterations were determined to be diagnostic in two separate individuals, thus, Kabuki syndrome was the most common diagnosis.

In 6 cases out of the full cohort (*n* = 27), parental consanguinity was recorded. The diagnostic yield was increased in cases with recorded consanguinity (3/6; 50%) as compared with those without consanguinity (7/21; 33%); however this was not statistically significant (Fisher’s exact *p* value = 0.6382). The types of diagnoses did differ however, with all three diagnoses in the consanguineous cases being biparentally inherited homozygous recessive variants, compared with none of the diagnoses in nonconsanguineous cases (Fisher’s exact *p* value = 0.0083).

There were no variants determined to be pathogenic/likely pathogenic but without relevance for the presenting fetal phenotype. In total, ten VUS were identified relating to six individual probands that were determined by the CRP to have no relevance to the fetal phenotype in each case (see Supplementary Table 2). Ethical approval for the study restricted the disclosure of genetic findings to those that were relevant to the FSA and thus these findings were not reported.

## Discussion

The genetic contribution to FSA is incompletely understood,^22^ and evidence relating to the clinical utility of next-generation sequencing (NGS) in the investigation of perinatal loss and morbidity in fetuses with structural anomalies is limited. Relatively small cohort studies have been published and indicate that the use of NGS in the perinatal context gives a diagnostic yield of 12–57% (refs. ^12,22–24^). However, such publications describe a heterogeneous mix of prenatal ultrasound diagnoses and autopsy findings in fetuses with congenital structural anomalies. A recent study investigating FSA described the use of prenatal samples negative for karyotyping and CMA consented for ES, once a parental decision on termination of pregnancy (TOP) was made. Of the 15 fetuses studied, ES identified pathogenic variants in 7 (47%) cases providing a likely causative diagnosis.^25^ The largest reported series to date performed ES in 84 deceased fetuses with structural anomalies, 52 of which comprised parental/fetus trios or quads. Where trio data were obtained a diagnostic yield of 24% was made. In those probands with only fetal DNA tested, there was a lower diagnostic rate of 14% (ref. ^12^). In a cohort of perinatal mortality cases (*n* = 50) with limited availability of proband DNA, exome sequencing of parental DNA was performed to identify heterozygous rare variants under a shared model of inheritance.^26^ Utilization of this novel strategy to diagnose recessive monogenic disorders in parents (subsequently confirmed as cosegregating in the affected fetus by targeted testing) demonstrated pathogenic/likely pathogenic variants in 24 different genes in 26/50 couples (52%). Where two or more fetuses were affected a genetic diagnosis was identified in 18/29 couples (62%), leading the authors to conclude that this is a powerful approach with high clinical utility for genetic diagnosis of lethal or prenatal-onset recessive conditions. Our reported diagnostic yield of 37% demonstrates further the value of trio ES in combination with a detailed autopsy.

An underlying genetic etiology for the congenital structural anomalies cannot usually be predicted accurately based on the prenatal USS appearances alone.^23^ This is primarily because the phenotype in the developing fetus is often inadequately defined. For example, a recent retrospective study examined the diagnostic utility and limitations of ES in prenatal cases with structural birth defects. DNA from 20 trios (fetal and parental), with normal karyotype and CMA findings, underwent ES and variant interpretation.^27^ The ES results were later reevaluated utilizing details of prenatal and postnatal phenotyping. Initial analysis using only a clinical description of prenatal ultrasound findings revealed no pathogenic/likely pathogenic variants in the 20 pregnancies evaluated but reanalysis with a combination of prenatal and postnatal phenotyping yielded pathogenic variants in at least 20% of cases.

In our series there was generally a very good association between prenatal USS descriptions and autopsy findings (as previously noted in a larger study from our group^6^). However in four cases (Fig. 2), subtle findings identified on autopsy led to a reclassification of the fetal phenotype. Though such findings do not necessarily influence the working clinical diagnosis (and as appertains to this cohort did not translate into an increased incidence of pathogenic variants), additional information available from autopsy can aid interpretation of ES detected variants and increase diagnostic yield or help exclude variants that come through the bioinformatics filtering process but are unlikely to account for the relevant FSA phenotype. In case 8 a homozygous missense substitution in *RAPSN* (c.485A>G p.(Glu162Gly) was identified in a fetus with a prenatal USS finding of fetal akinesia deformation sequence and autopsy confirmed the presence features of arthrogryposis multiplex. *RAPSN* encodes the receptor-associated protein, rapsyn (RAPSN) involved in AChR localization and assembly and localization of the postsynaptic muscle nicotinic acetylcholine receptor. Recessively inherited pathogenic variants in *RAPSN* were initially described in individuals with congenital myasthenic syndrome^28^ and subsequently with lethal fetal akinesia.^29^ Previously it has been noted that early severe onset disease (fetal akinesia/multiple pterygium syndrome) tended to be associated with severe loss-of-function pathogenic variants and later-onset myasthenic syndrome with missense substitutions causing milder alterations of function.^29,30^ Though *RAPSN* missense substitutions (as case 8) have been associated with severe disease the availability of detailed information from the autopsy (including lack of evidence for central nervous system cause) contributed to a consensus classification of the variant as likely pathogenic. In addition the combination of ES and a more detailed autopsy phenotype can expand knowledge of the prenatal phenotype of developmental disorders and/or rarer manifestations of severe cases. For example, case 25 was found to have renal cystic dysplasia on prenatal ultrasonography and also demonstrated polymicrogyria of the central nervous system with calcification. ES analysis revealed a homozygous truncating *CPT2* variant consistent with a diagnosis of carnitine palmitoyltransferase (CPT) II deficiency. Although this rare disorder typically presents with myoglobinuria in adults or fasting hypoglycemia in children, rarely severe cases may present prenatally.^31^ In a review of 19 such cases, cerebral calcification was noted in 3 cases and polymicrogyria in 1 case.^31^ In cases in which ES was not diagnostic, such as case 23, autopsy examination identified abnormalities that were not identifiable on prenatal ultrasound and this refined the phenotypic abnormalities and might facilitate future diagnosis. It is acknowledged, however, that this cohort of cases were highly selected with a degree of selection bias.

The genetic findings greatly assist assessment of recurrence risk in future pregnancies. In the four cases with de novo findings it was possible to reassure the parents of a low recurrence risk. The parents with inherited diagnoses (6) were counseled as to the likely risk of recurrence according to the mode of inheritance in each individual case (case studies 1 and 2). Those with recurrence risks then had the option for invasive prenatal diagnosis in a subsequent pregnancy to ascertain if the fetus was affected with the same condition.

For the duration of this study the trio samples were reanalyzed at five planned time points (data freezes) to include recently identified genes, as well as new alterations in known genes that associate with congenital malformation (Supplementary Table 1). This resulted in the detection of pathogenic variants in two fetuses that previously lacked a genetic diagnosis, in case 25 (*CPT2* stop gained mutation) and case 11 (*DNAH5* compound heterozygous frameshift/stop gained mutations). Both genes were added into the virtual gene panel after the initial review of the fetuses, demonstrating the strength of our iterative analysis approach. The added value of systematic review of ES data in the light of new knowledge has been shown to significantly improve diagnostic yield in the context of the Deciphering Developmental Disorders study.^17^ Through improved variant calling methods, novel variant detection algorithms, updated variant annotation, evidence-based filtering strategies, and newly discovered disease-associated genes, diagnostic yield in a pediatric cohort with clinically diagnosed development delay was increased by 13% (from 27% to 40%). This raises the importance of reanalysis of perinatal sequencing data in relation to ongoing genetic diagnosis, and demonstrates the need for further research as to the potential added value of genomic reanalysis in undiagnosed fetal and neonatal mortality cases.

Our findings therefore support the routine use of molecular autopsy using trio ES and full autopsy to investigate perinatal loss and elucidate the underlying genetic basis of structural developmental abnormality, particularly in relation to severe “lethal” fetal phenotypes where the ethical issues that cause concern with testing in an ongoing pregnancy are somewhat ameliorated in pregnancies that have already ended.

## Electronic supplementary material

Supplementary Information

Case Study 1

Case Study 2
