# Supplementary material for: Molecular autopsy by trio exome sequencing (ES) and postmortem examination in fetuses and neonates with prenatally identified structural anomalies
Source: Genet Med. 2018 Oct 8;21(5):1065–73. doi: 10.1038/s41436-018-0298-8 (PMC6752266; doi:10.1038/s41436-018-0298-8)
Supplement: Supplementary file 1 — Supplementary Information [file 41436_2018_298_MOESM1_ESM.docx]

**Supplementary Information**

**Supplementary Methods**

*Exome sequencing, variant detection and annotation:* After receipt of DNA samples at the Wellcome Sanger Institute for WES genomic DNA (~125ng) was fragmented to an average size of 150 base pairs (bp) and DNA libraries were prepared and processed using standard Illumina methodologies. A portion of each library was used to create an equimolar pool comprising 8 indexed libraries. For each pool exome capture was undertaken with the Agilent SureSelect XT Human All Exon V5 Plus with custom ELID#0337431 (Agilent Technologies, Santa Clara, CA, USA) and analysed by 75-base paired-end sequencing (with 6 samples per lane on Illumina HiSeq 2500) following the manufacturer’s instructions.

Mapping of short-read sequences was performed with the Burrows-Wheeler aligner (BWA; version 0.59) [41](https://www.nature.com/nature/journal/v542/n7642/full/nature21062.html#ref41) backtrack algorithm with the GRCh37 1000 Genomes Project phase 2 reference (also known as hs37d5). SNPs and indels were identified from CRAM data using GATK HaplotypeCaller version 3.6 (Van der Auwera et al. 2013). DeNovoGear (Ramu et al. 2013) was used to identify candidate *de novo* variants. Copy number variations (CNVs) were detected using CoNVex (<http://www.uk10k.org/assets/ashg_vijayarangakannan_etal_2012.pdf>), and inheritance predicted with CIFER (https://github.com/jeremymcrae/cifer). Outputs from GATK haplotype caller, DeNovoGear and CoNVex were merged into a single variant call format (VCF) file per trio and annotated with allele frequencies from the Exome Aggregation Consortium (ExAC) (Lek et al. 2016) , the 1000 Genomes Project (Genomes Project et al. 2015), UK10K (Consortium et al. 2015), the Exome Sequencing Project (ESP) (Exome Variant Server, NHLBI GO Exome Sequencing Project (ESP), Seattle, WA (URL: http://evs.gs.washington.edu/EVS/) [accessed Jan 2013], and from unaffected parents from the Deciphering Developmental Disorders (DDD)(Wright et al. 2015) and PAGE studies. Ensembl’s Variant Effect Predictor (VEP version 85) (McLaren et al. 2016) was used to annotate the predicted effect of each variant. UPD was identified using UPDio (King et al. 2014). The bioinformatics pathway is summarised in supplementary Figure 2.

Quality control was performed on all VCF files to exclude samples where exome sequencing was of poor quality and to exclude potentially contaminated samples. KING (Manichaikul et al. 2010) and PC-Relate (Conomos et al. 2016) were used to confirm that the identity of the trios was correct and that no sample swaps had occurred.

Variants were filtered to identify those of potential clinical significance. Protein-altering variants were selected using VEP annotation and common variants were excluded using minor allele frequencies (including ExAC, DDD unaffected parents, ESP, UK10K and the 1000 genomes project). A developmental disorder-associated gene list ([www.ebi.ac.uk/gene2phenotype](http://www.ebi.ac.uk/gene2phenotype); downloaded in April 2017) (Wright et al. 2015) was modified to exclude genes without a prenatal phenotype, and supplemented with 116 genes identified from the literature and deemed to be robustly associated with a prenatal phenotype (Supplementary Table 1). Rare, protein altering variants in genes in this list where the inheritance pattern of the variant matches that of the gene were selected for clinical review.

**Supplementary Table 1:** Genes identified from the literature relating to conditions with potentially prenatally manifesting phenotypes, added to 1421 developmental disorder genes included in the DDG2P panel ([www.ebi.ac.uk/gene2phenotype](http://www.ebi.ac.uk/gene2phenotype)) (downloaded 25/4/17).

| MYBPC1 |
| --- |
| COL6A2 |
| MSH2 |
| MLH1 |
| MSH6 |
| TPM3 |
| TNXB |
| COL1A2 |
| PIEZO1 |
| DSP |
| ASNS |
| EMG1 |
| FBLN5 |
| GPI |
| CYP17A1 |
| CYP21A2 |
| CYP11A1 |
| CDAN1 |
| POR |
| OCLN |
| LZTFL1 |
| KCNJ1 |
| SLC12A1 |
| PRG4 |
| SLC26A3 |
| CRLF1 |
| CPT2 |
| LTBP4 |
| ATP6V0A2 |
| CFTR |
| INSR |
| G6PC3 |
| ITGA6 |
| ITGB4 |
| MUSK |
| GRIP1 |
| ADAMTSL2 |
| GBE1 |
| SP110 |
| AP3B1 |
| KISS1R |
| ABCA12 |
| TGM1 |
| TCTN1 |
| TMEM138 |
| TMEM231 |
| AGPAT2 |
| PTRF |
| IER3IP1 |
| B3GAT3 |
| CHKB |
| SGCA |
| SEPN1 |
| AGRN |
| CHAT |
| KLHL41 |
| LMOD3 |
| TNNT1 |
| CEP164 |
| INVS |
| PPIB |
| CRTAP |
| OSTM1 |
| TCIRG1 |
| B3GALTL |
| DNAAF1 |
| DNAH11 |
| DNAH5 |
| DNAI1 |
| HSD17B3 |
| SRD5A2 |
| PKLR |
| ACE |
| REN |
| IQCB1 |
| CHRND |
| NHEJ1 |
| TTC21B |
| SOST |
| SMN1 |
| HES7 |
| LIFR |
| TBC1D20 |
| ADAMTS10 |
| EMD |
| UBA1 |
| ACTG2 |
| LMBR1 |
| CYP11B1 |
| KCNJ2 |
| TNNI2 |
| SUFU |
| RPS26 |
| RPS17 |
| RPL5 |
| RPL11 |
| RPS10 |
| COL5A1 |
| COL5A2 |
| TGIF1 |
| PROK2 |
| PKD1 |
| PKD2 |
| TBX6 |
| H19 |
| PROKR2 |
| FGF8 |
| NR0B1 |
| ABCC8 |
| KAL1 |
| MBTPS2 |
| ZNF423 |
| ARL13B |
| POMK |
| ADAMTS17 |
| EPHB4 |

**Supplementary Table 2:** Information on all variants reviewed by the clinical review panel (CRP) and review outcomes. Coordinates given in hg19.

| Patient number | Phenotypic Class | Chr | Position | Ref | Alt | Gene | Variant Type | Inheritance | Zygosity | Consequence | SIFT | PolyPhen | gnomAD_AF | CADD_PHRED | pLI (gene) (Lek et al. 2016) | missense constraint Z-score (gene) (Lek et al. 2016) | Review outcome |
| --- | --- | --- | --- | --- | --- | --- | --- | --- | --- | --- | --- | --- | --- | --- | --- | --- | --- |
| 1 | FADS | 13 | 32914723 | G | C | BRCA2 | missense_variant | Inherited | Homozygous | ENST00000544455:p.2077_K/N | deleterious(0) | possibly_damaging(0.502) | 0.000119 | 24.3 | 0 | -4.71 | Not relevant |
| 1 | FADS | 21 | 46893848 | G | A | COL18A1 | missense_variant | Inherited | Compound heterozygous | ENST00000355480:p.411_V/M | deleterious(0.03) | possibly_damaging(0.857) | 0.001017 | 22.3 | 0 | -1.62 | Not relevant |
| 1 | FADS | 21 | 46925140 | C | CGGCCCCCCA | COL18A1 | inframe_insertion | Inherited | Compound heterozygous | ENST00000355480:p.1167-1168_-/GPP | NA | NA | 0.0007809 | 13.54 | 0 | -1.62 | Not relevant |
| 4 | Skeletal | X | 38515292 | G | A | TSPAN7 | missense_variant | Inherited from unaffected mother (X) | Heterozygous | ENST00000480976:p.42_G/E | - | unknown(0) | - | 5.461 | 0.87 | 1.63 | VOUS, not relevant |
| 5 | Cardiac | 10 | 15655740 | G | A | ITGA8 | missense_variant | Inherited | Compound heterozygous | ENST00000378076:p.491_A/V | deleterious(0) | possibly_damaging(0.865) | - | 25.7 | 0 | -1.25 | VOUS, not relevant |
| 5 | Cardiac | 10 | 15614323 | C | T | ITGA8 | missense_variant | Inherited | Compound heterozygous | ENST00000378076:p.842_V/M | deleterious(0) | probably_damaging(1) | 0.0003209 | 33 | 0 | -1.25 | VOUS, not relevant |
| 8 | FADS | 1 | 35251013 | GCCTGCACAAGGA | G | GJB3 | inframe_deletion | Inherited | Homozygous | ENST00000373366:p.217-221_GLHKD/G | - | - | 0.0002641 | 7.047 | 0 | -0.81 | VOUS, not relevant |
| 13 | Genitourinary | 19 | 47259533 | C | A | FKRP | missense_variant | Inherited | Homozygous | ENST00000318584:p.276_L/I | tolerated(0.06) | benign(0.3) | 0.001002 | 22.6 | 0 | 2.65 | Not reportable (secondary finding) |
| 18 | Multisystem | 2 | 216176968 | G | A | ATIC | missense_variant | Inherited | Compound heterozygous | ENST00000427397:p.35_G/S | - | unknown(0) | - | 7.996 | 0 | -1.27 | VOUS, not relevant |
| 18 | Multisystem | 2 | 216214000 | T | C | ATIC | stop_lost | Inherited | Compound heterozygous | ENST00000426233:p.232_*/Q | - | - | 2.44E-05 | 6.758 | 0 | -1.27 | VOUS, not relevant |
| 25 | Multisystem | 1 | 53662643 | T | TAGCAAG | CPT2 | stop_gained | Inherited | Homozygous | ENST00000371486:p.10_W/*QG | - | - | - | - | 0 | -0.45 | Diagnostic |
| 11 | Cardiac | 5 | 13701425 | T | TA | DNAH5 | frameshift_variant | Inherited | Compound heterozygous | ENST00000265104:p.4486-4487_-/X | - | - | 5.28E-05 | 37 | 0 | -1.75 | Diagnostic |
| 11 | Cardiac | 5 | 13820533 | G | A | DNAH5 | stop_gained | Inherited | Compound heterozygous | ENST00000265104:p.2255_R/* | - | - | 1.63E-05 | 40 | 0 | -1.75 | Diagnostic |
| 9 | FADS | 13 | 103524633 | C | CA | ERCC5 | frameshift_variant | Inherited | Homozygous | ENST00000355739:p.922_Q/QX | - | - | 3.66E-05 | 35 | 0 | -0.61 | Diagnostic |
| 21 | Hydrops fetalis | X | 49107902 | G | A | FOXP3 | missense_variant | Inherited from unaffected mother (X) | Heterozygous | ENST00000376207:p.397_R/W | deleterious(0) | probably_damaging(0.977) | 5.62E-06 | 33 | 0.95 | 0.86 | Diagnostic |
| 3 | Multisystem | 12 | 49444122 | G | T | KMT2D | stop_gained | De novo | Heterozygous | ENST00000301067:p.1083_C/* | - | - | - | 35 | 1 | 3.1 | Diagnostic |
| 22 | Multisystem | 12 | 49422652 | C | A | KMT2D | stop_gained | De novo | Heterozygous | ENST00000301067:p.4781_E/* | - | - | - | 56 | 1 | 3.1 | Diagnostic |
| 16 | Multisystem | 18 | 59821771 | CACAAACCT | C | PIGN | splice_donor_variant | Inherited | Compound heterozygous | ENST00000357637:p.183-? | - | - | 0.0001277 | 35 | 0 | -2 | Diagnostic |
| 16 | Multisystem | 18 | 59815467 | A | C | PIGN | missense_variant | Inherited | Compound heterozygous | ENST00000357637:p.218_H/Q | deleterious(0) | probably_damaging(0.989) | 2.61E-05 | 25.6 | 0 | -2 | Diagnostic |
| 24 | Brain | 19 | 18273784 | G | A | PIK3R2 | missense_variant | De novo | Heterozygous | ENST00000222254:p.373_G/R | deleterious(0) | probably_damaging(1) | - | 27.4 | 0.94 | 3.03 | Diagnostic |
| 8 | FADS | 11 | 47469410 | T | C | RAPSN | missense_variant | Inherited | Homozygous | ENST00000298854:p.162_E/G | deleterious(0) | benign(0.408) | 8.21E-06 | 26.1 | 0.03 | -0.08 | Diagnostic |
| 10 | Multisystem | 1 | 155874263 | T | C | RIT1 | missense_variant | De novo | Heterozygous | ENST00000368322:p.107_M/V | deleterious(0) | probably_damaging(0.972) | - | 24.6 | 0.67 | 2.2 | Diagnostic |

Conomos MP, Reiner AP, Weir BS, Thornton TA. 2016. Model-free Estimation of Recent Genetic Relatedness. *Am J Hum Genet* **98**: 127-148.

Consortium UK, Walter K, Min JL, Huang J, Crooks L, Memari Y, McCarthy S, Perry JR, Xu C, Futema M et al. 2015. The UK10K project identifies rare variants in health and disease. *Nature* **526**: 82-90.

Genomes Project C, Auton A, Brooks LD, Durbin RM, Garrison EP, Kang HM, Korbel JO, Marchini JL, McCarthy S, McVean GA et al. 2015. A global reference for human genetic variation. *Nature* **526**: 68-74.

King DA, Fitzgerald TW, Miller R, Canham N, Clayton-Smith J, Johnson D, Mansour S, Stewart F, Vasudevan P, Hurles ME et al. 2014. A novel method for detecting uniparental disomy from trio genotypes identifies a significant excess in children with developmental disorders. *Genome Res* **24**: 673-687.

Lek M, Karczewski KJ, Minikel EV, Samocha KE, Banks E, Fennell T, O'Donnell-Luria AH, Ware JS, Hill AJ, Cummings BB et al. 2016. Analysis of protein-coding genetic variation in 60,706 humans. *Nature* **536**: 285-291.

Manichaikul A, Mychaleckyj JC, Rich SS, Daly K, Sale M, Chen WM. 2010. Robust relationship inference in genome-wide association studies. *Bioinformatics* **26**: 2867-2873.

McLaren W, Gil L, Hunt SE, Riat HS, Ritchie GR, Thormann A, Flicek P, Cunningham F. 2016. The Ensembl Variant Effect Predictor. *Genome Biol* **17**: 122.

Ramu A, Noordam MJ, Schwartz RS, Wuster A, Hurles ME, Cartwright RA, Conrad DF. 2013. DeNovoGear: de novo indel and point mutation discovery and phasing. *Nat Methods* **10**: 985-987.

Van der Auwera GA, Carneiro MO, Hartl C, Poplin R, Del Angel G, Levy-Moonshine A, Jordan T, Shakir K, Roazen D, Thibault J et al. 2013. From FastQ data to high confidence variant calls: the Genome Analysis Toolkit best practices pipeline. *Curr Protoc Bioinformatics* **43**: 11 10 11-33.

Wright CF, Fitzgerald TW, Jones WD, Clayton S, McRae JF, van Kogelenberg M, King DA, Ambridge K, Barrett DM, Bayzetinova T et al. 2015. Genetic diagnosis of developmental disorders in the DDD study: a scalable analysis of genome-wide research data. *Lancet* **385**: 1305-1314.

**Supplementary Figure 1**

**
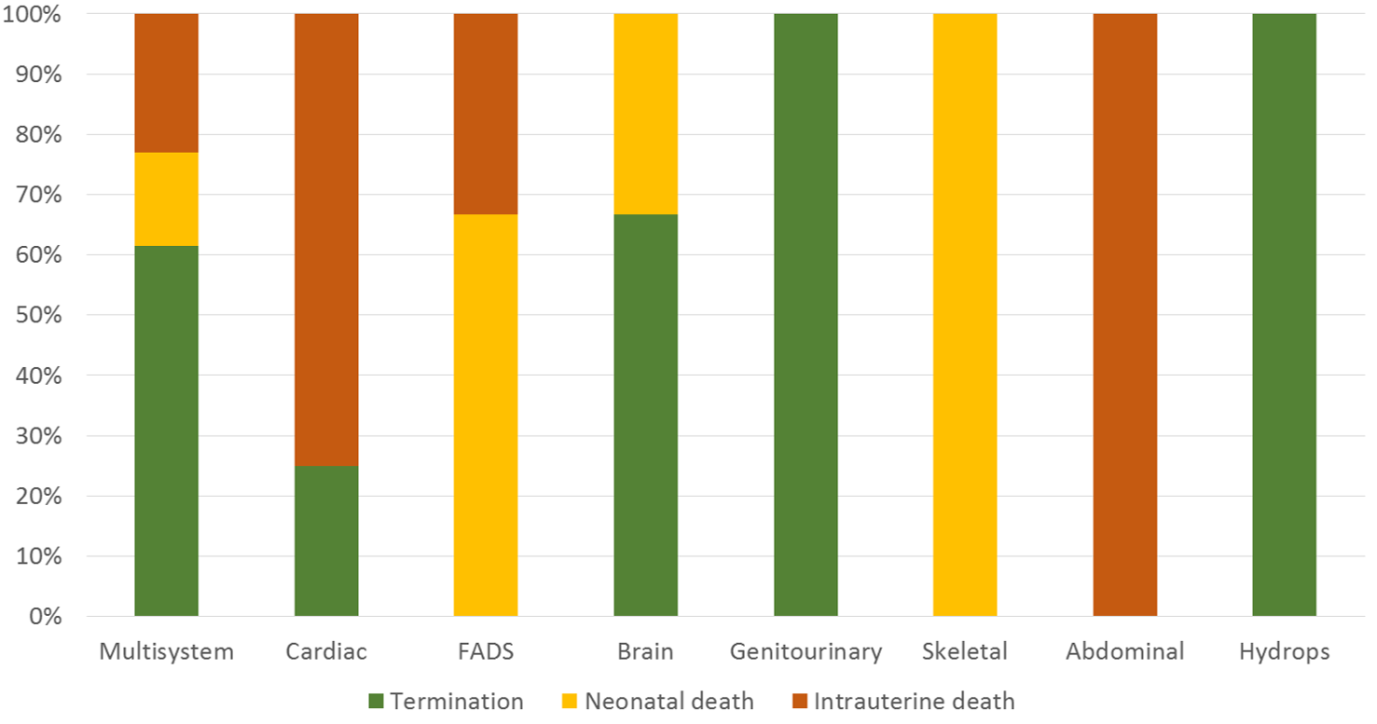
**

**Figure to show pregnancy outcome per proband according to phenotype class determined at post-mortem examination**
