# Supplementary material for: Molecular autopsy by trio exome sequencing (ES) and postmortem examination in fetuses and neonates with prenatally identified structural anomalies
Source: Genet Med. 2018 Oct 8;21(5):1065–73. doi: 10.1038/s41436-018-0298-8 (PMC6752266; doi:10.1038/s41436-018-0298-8)

**Case Study 1 (Patient Number 3)**

*Clinical background:*

G4 P2: +1 (2013 – spontaneous miscarriage at 16 weeks with post-mortem; 2014 – NVD of healthy male at 38 weeks; 2015 – LSCS of stillborn male at 35 weeks with post-mortem). The parents were consanguineous (second cousins) of Pakistani ethnicity, and the mother was known to be a carrier of alpha-thalassaemia. Ultrasound examination at 26+5 weeks showed minimal fetal movements with severely abnormal posture, bilateral hydrothoraces (mild), and appearances of Fetal Akinesia Deformation Sequence (FADS) similar to the previous stillborn baby. QF-PCR for common aneuploidy and CMA for copy number variation was undertaken and reported as normal. The parents opted for TOP at 32 weeks and a post-mortem was performed which confirmed FADS.

*Whole exome sequencing (WES) variant data:*

WES and bioinformatic filtering identified a homozygous 1bp insertion within exon 13 of the *ERCC5* gene (NM_000123.3:c.2766dupA) in the fetus, predicted to give a frameshift effect, introducing a premature termination codon (NP_000114.2:p.Leu923ThrfsTer7). WES data indicated both parents were heterozygous carriers of the variant.

*Clinical Review Panel (CRP) interpretation:*

Variants in *ERCC5* (encoding a DNA endonuclease involved in DNA excision repair (https://ghr.nlm.nih.gov/gene/ERCC5) are associated with cerebrooculofacioskeletal (COFS) syndrome (MIM 616570), with arthrogryposis, microcephaly, and intrauterine growth restriction noted as prenatal manifestations (reference Drury 2014 and/or OMIM https://www.omim.org/entry/616570#1). The variant is present in gnomAD (reference - http://gnomad.broadinstitute.org/) with a frequency of 3.655e-5, corresponding to 9 heterozygous carriers, and no instances of the variant in a homozygous state. This same variant had been previously reported once in association with a severe manifestation of cerebrooculofacioskeletal (COFS) syndrome presenting in the prenatal period (Drury et al. 2014 Am J Med Genet Part A 164A:1777-1783). Given the rarity of the variant, the severity of the likely functional impact, the prior report of the variant as pathogenic, and the degree of concordance between the phenotype of the fetus and the manifestations associated with the gene, the variant was classed as clearly pathogenic, with full contribution to the phenotype according to ACMG guidelines. The variant was validated using bi-directional Sanger sequencing and reported to the clinical geneticist overseeing the care of the family. The parents therefore carried a 1:4 recurrence risk as a couple in any future pregnancy.

*Clinical utility:*

The parents were informed of the result at a subsequent clinic appointment with their clinical geneticist. During this discussion the mother informed the clinical team that she was currently in the early stages of pregnancy. The parents opted for prenatal diagnosis by CVS to assess for the same mutation in the fetus. Sequence analysis of fetal DNA extracted from the CVS detected homozygosity for the familial pathogenic variant c.2766dupA p.(Leu923Thrfs*7) in exon 13 of the *ERCC5* gene. Microsatellite marker analysis found no significant evidence of maternal cell contamination in the CVS sample. The result indicated that the fetus was highly likely to be affected with an *ERCC5*-related disorder. The parents opted for early second trimester TOP following this diagnosis.

*Protein schematic of ERCC5 gene taken from DECIPHER highlighting variants deposited in ExAC, gnomAD, ClinVar and DECIPHER and location of p.(Leu923Thrfs*7) (date 15.03.2018)*


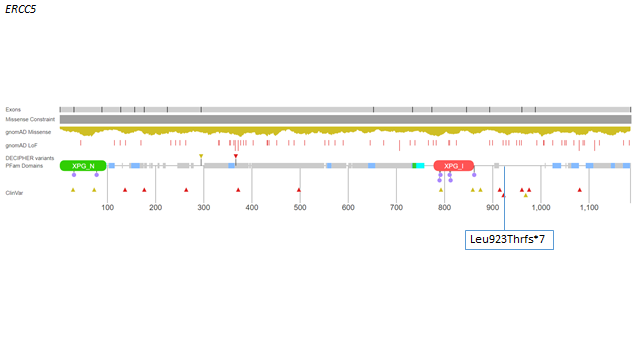

Supplement: Supplementary file 2 — Case Study 1 [file 41436_2018_298_MOESM2_ESM.docx]
