# Supplementary material for: Molecular autopsy by trio exome sequencing (ES) and postmortem examination in fetuses and neonates with prenatally identified structural anomalies
Source: Genet Med. 2018 Oct 8;21(5):1065–73. doi: 10.1038/s41436-018-0298-8 (PMC6752266; doi:10.1038/s41436-018-0298-8)
Supplement: Supplementary file 3 — Case Study 2 [file 41436_2018_298_MOESM3_ESM.docx]

**Case Study 2 (Patient Number 7)**

*Clinical background:*

G3 P1: +1 (1997 – TOP, 2008 – NVD healthy female at 37 weeks). Non-consanguineous parents of Caucasian ethnicity with a family history of multiple miscarriages (patient’s mother and maternal aunt). Ultrasound examination at 20 weeks revealed severe hydrops fetalis. QF-PCR and CMA analysis were undertaken and excluded common aneuploidy and variations in copy number. The parents opted for TOP at 20+3 weeks and a post-mortem examination was performed which confirmed fetal hydrops with consequent pulmonary hypoplasia.

*Whole exome sequencing (WES) variant data:*

WES and bioinformatic filtering revealed a hemizygous C>T variant (NM_014009.3:c.1189C>T) in the gene *FOXP3* in the male fetus, giving a missense change in the resultant protein’s primary sequence (NP_054728.2:p.Arg397Trp). WES data from the parents indicated the X chromosome variant had been inherited from the mother who is a heterozygous carrier of the variant.

*Clinical Review Panel (CRP) interpretation:*

Variants in the gene *FOXP3* (encoding a transcription factor involved in immune regulation (reference - https://ghr.nlm.nih.gov/gene/FOXP3)) are associated with immunodysregulation, polyendocrinopathy, and enteropathy, X-linked (IPEX). The variant identified was predicted to be damaging by both SIFT ^[1]^ and Polyphen2 ^[2]^. The variant was not present in any population database (dbSNP, ESP, ExAC), and had previously been reported in association with immunodysregulation polyendocrinopathy enteropathy X-linked (IPEX) syndrome in two previous families (Wildin *et al*. 2001 Nat Genet. 27:18-20; Xavier-da-Silva *et al*. 2015 Clin Immunol. 159:131-140). *In silico* and *in vitro* functional studies are supportive of this variant having a detrimental effect on protein function (Lopes *et al*. 2006 J Immunol. 177:1311-3142; Andersen *et al*. 2012 Front Immunol. 3(10)). The variant was determined to be causally related to prenatal phenotype of hydrops fetalis and was thus validated by Sanger sequencing and reported to the clinical geneticist involved with the care of the family. The parents therefore carried a 1:2 risk of recurrence in any future male pregnancy.

*Clinical utility:*

The parents were informed of the result at a subsequent clinical appointment by the clinical geneticist. The mother had since experienced another TOP at 20 weeks for the same prenatal phenotype. Carrier testing for relatives (mother, aunt and sister) was subsequently offered to confirm segregation of the variant within the family, and potentially guide future prenatal diagnosis for at risk family members.

Protein schematic of FOXP3 gene taken from DECIPHER highlighting variants deposited in ExAC, gnomAD, ClinVar and DECIPHER and location of p.Arg397Trp (date 15.03.2018)


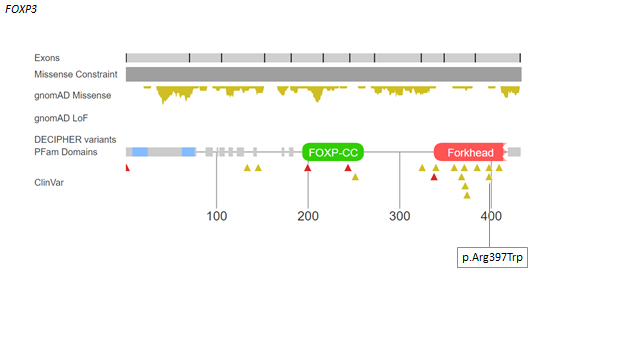


*References:*

[1] Kumar P, Henikoff S, Ng PC. Predicting the effects of coding non-synonymous variants on protein function using the SIFT algorithm. *Nat Protoc. 2009*;4(7):1073-81

[2] Adzhubei IA, Schmidt S, Peshkin L, Ramensky VE, Gerasimova A, Bork P, Kondrashov AS, Sunyaev SR. Nat Methods 7(4):248-249 (2010).
